# Antidiabetic Potential of Sea Urchin *Tripneustes gratilla* Nanosuspension Based on In Vitro Enzyme Inhibition, In Vivo Evaluation, and Chemical Profiling Approaches

**DOI:** 10.3390/cimb48010008

**Published:** 2025-12-21

**Authors:** Ahmed K. B. Aljohani, Aryam S. Alharbi, Asalah B. Alhazmi, Manhal N. Hudhayri, Israa B. Almuwallad, Maya A. Alhazmi, Shuruq M. Almohammadi, Atheer I. Alsaleh, Ahmed Aldhafiri, Heba M. Eltahir, Mekky M. Abouzied, Hamad Alrbyawi, Mohamed S. Mohamed, Mahran Mohamed Abdel-Emam, Fahd M. Abdelkarem

**Affiliations:** 1 Department of Pharmacognosy and Pharmaceutical Chemistry, College of Pharmacy, Taibah University, Madinah 42353, Saudi Arabia; akjohani@taibahu.edu.sa; 2 Health and Life Research Center, Taibah University, Madinah 42353, Saudi Arabia; 3 College of Pharmacy, Taibah University, Madinah 42353, Saudi Arabia; aryamsaadalharbi@gmail.com (A.S.A.); alhazmiasalh@gmail.com (A.B.A.); manhal.n.h@gmail.com (M.N.H.); esraaal17320@gmail.com (I.B.A.); mayaalhazmi3@gmail.com (M.A.A.); shuruq.m.a.almohammadi@gmail.com (S.M.A.); atheer_1331@hotmail.com (A.I.A.); 4 Department of Pharmacology and Toxicology, College of Pharmacy, Taibah University, Madinah 42353, Saudi Arabia; adhafiri@taibahu.edu.sa (A.A.); htahir@taibahu.edu.sa (H.M.E.); mabouzied@taibahu.edu.sa (M.M.A.); 5 Department of Pharmaceutics and Pharmaceutical Industries, College of Pharmacy, Taibah University, Madinah 42353, Saudi Arabia; hrbyawi@taibahu.edu.sa; 6 Department of Pharmaceutics and Pharmaceutical Technology, Faculty of Pharmacy, Al-Azhar University, Assiut 71524, Egypt; mohamedsabry@azhar.edu.eg; 7 Department of Biochemistry, Faculty of Veterinary Medicine, Zagazig University, Zagazig 44511, Egypt; mahranmohamed1234@gmail.com; 8 Department of Pharmacognosy and Medicinal Plants, Faculty of Pharmacy, Al-Azhar University, Assiut 71524, Egypt

**Keywords:** sea urchin, antioxidant, α-amylase, α-glucosidase, antidiabetic

## Abstract

Diabetes mellitus represents one of the main health challenges worldwide, characterized by hyperglycemia and long-term serious microvascular and macrovascular complications. Marine organisms are a promising reservoir of bioactive metabolites for developing effective antidiabetic therapies with fewer side effects. The sea urchin *Tripneustes gratilla* (*T. gratilla*) is widely distributed in the Red Sea, with limited reports of its pharmacological activities and chemical characterization. In this study, a nanosuspension formulation of *T. gratilla* extract (*T. gratilla*-NS) was developed to enhance the bioavailability of its bioactive constituents. This study investigated the antidiabetic potential of *T. gratilla* extract through an integrated approach encompassing chemical profiling of the extract, assessment of its alcoholic extract for in vitro inhibitory effects on α-amylase and α-glucosidase, and in vivo evaluation of *T. gratilla*-NS in an alloxan-induced diabetic rat model. We found that the alcoholic extract showed potent inhibitory action toward α-amylase with IC_50_ 5.31 ± 0.05 µg/mL and moderate inhibitory activity toward α-glucosidase with IC_50_ 21.36 ± 0.06 µg/mL. *T. gratilla*-NS significantly increased insulin levels, reduced blood glucose levels, and restored pancreatic damage. Furthermore, it enhanced the levels of superoxide dismutase and total antioxidant capacity with a concomitant decrease in malondialdehyde concentration in pancreatic tissue. The observed activities could be attributed to a wide array of diverse compounds, terpenes, mainly sesquiterpenes, diterpenes, steroids, and polyunsaturated fatty acids detected by GC-MS, compounds with a phenolic nucleus equal to 54.26 ± 1.27 mg. GAE/g of extract. This research highlights the dual role of *T. gratilla*-NS in combating diabetes and subsequently attenuating its associated complications.

## 1. Introduction

Diabetes mellitus is a metabolic disorder characterized by persistent hyperglycemia and represents a major global public health challenge due to its long-term complications, increasing prevalence, and economic burden on individuals and healthcare systems [1]. Poorly controlled diabetes can lead to a wide spectrum of complications affecting multiple organs and systems, with oxidative stress and inflammation playing pivotal roles in disease progression. Microvascular complications include neuropathy, retinopathy, and nephropathy, whereas macrovascular complications encompass atherosclerosis, myocardial infarction, stroke, and peripheral artery disease, sometimes progressing to amputation and long-term disability [2,3]. At the national level, the growing costs of managing diabetes and its complications impose significant socio-economic burdens, highlighting the importance of research focused on prevention, treatment, and complication control from both medical and economic perspectives [4,5].

In the Middle East, diabetes is a serious disorder with a higher prevalence rate globally [6]. Strategies for diabetes control are based on lifestyle modification and regular physical activity, together with pharmacological therapies, which reduce postprandial glucose, increase insulin secretion, or increase tissue sensitivity, or combinations based on disease severity. Synthetic antidiabetic agents are usually accompanied by adverse effects such as hypoglycemia, hepatotoxicity, and complications with long-term use, in addition to the need for increasing the dose in cases with low absorption [7,8]. Natural products derived from alternative sources such as marine organisms represent an excellent option to overcome drug limitations and provide a multi-targeted strategy for diabetes management [9,10,11].

Sea urchins are tiny organisms belonging to the phylum Echinodermata and distributed in seas and oceans, with approximately 800 species identified to date [12]. They play an essential role in the coral reef ecosystem by grazing on algae. Urchin structures include gonads and non-edible spines, outer shells, Aristotle lanterns, and viscera [13]. Several metabolites, minerals, and vitamins were detected in different parts of sea urchins and were reported to exert diverse pharmacological activities such as anti-inflammatory, anticoagulant, antioxidant, anticancer, antiviral, and antimicrobial properties [12,13,14,15].

In the Red Sea regions, several sea urchins were detected with different sizes, such as *Echinomtera mathaei*, *Diadema setosum*, and *Tripneustes gratilla* (*T. gratilla*), and, despite there being few research reports, several metabolites such as steroids, terpenes, lipid derivatives, and sulfated compounds have been identified, and antiviral, anticancer, and antimicrobial activity has been reported [16,17,18]. *T. gratilla* inhabits tropical oceans and the Red Sea, possessing high-quality gonads and being among the most commercially harvested species in many countries [19]. Previous reports have demonstrated its antimicrobial activity with the isolation of novel peptides and antioxidant anti-inflammatory activities and its use in anti-poisoning drugs [20]. Despite its ecological abundance and recognized nutritional significance, the pharmacological profile of *T. gratilla*, particularly its antidiabetic potential, remains poorly explored.

Nanotechnology is an advanced technique used to overcome the challenges of solubility and bioavailability in pharmaceuticals. By formulating natural extracts in the form of nanoparticles, the bioactivity of such extracts can be substantially improved via increasing solubility and absorption, potentially allowing for lower doses and minimizing side effects [21]. Hence, a large number of research groups have focused on improving the formulation of drugs from marine invertebrates, mainly sea urchins, to overcome dissolution and bioavailability challenges [22,23].

In this study, the antidiabetic potential of a nanosuspension formulation of an alcoholic extract of *Tripneustes gratilla* (*T. gratilla-NS*) was assessed in vivo using a diabetic rat model by measuring insulin, glucose, and HOMA (Homeostatic Model Assessment) indices, along with evaluating antioxidant and oxidant markers. In parallel, the alcoholic extract was examined in vitro for its inhibitory effects on α-amylase and α-glucosidase, its chemical composition was characterized through GC–MS analysis, and phenolic content was quantified spectrophotometrically.

## 2. Materials and Methods

### 2.1. Collection and Extraction of T. gratilla

#### 2.1.1. Collection and Identification of *T. gratilla*

The sea urchin *T. gratilla* was collected at a depth of 1–3 m in the Red Sea near Hurghada city and identified by Professor Aldoushy Mahdy (Zoology Department, Faculty of Sciences, Al-Azhar University, Assiut, Egypt) and the specimen coded (Tg-23) was deposited in the Pharmacognosy Department, Faculty of Pharmacy, Al-Azhar University, Assiut, Egypt.

#### 2.1.2. Extraction of *T. gratilla*

Three hundred grams of the sea urchin *T. gratilla* was macerated in 70% ethanol (4 × 1 L) at room temperature until exhaustion. Then the extract was filtered, and the filtrate was concentrated using a rotary evaporator at 35 °C to yield a brown viscous residue, which was labeled as the total extract (21.2 g). The total extract was further chromatographed over Diaion HP-20 (Supelco analytical, Bellefonte, PA, USA) in order to remove salts using 100% H_2_O and 100% MeOH as a mobile phase to afford H_2_O (6.7 g) and alcoholic (13.9 g) extracts, respectively. The alcoholic extract was subjected to further assays to evaluate the antidiabetic potential of the sea urchin *T. gratilla*.

### 2.2. In Vitro Antidiabetic Assay

#### 2.2.1. α-Amylase-Inhibitory Activity

The α-amylase-inhibitory activity was evaluated using the 3,5-dinitrosalicylic acid (DNSA) method [24]. The extract of *T. gratilla* was initially dissolved in a minimal volume of 10% dimethyl sulfoxide (DMSO), followed by dilution with phosphate buffer (0.02 M Na_2_HPO_4_/NaH_2_PO_4_, containing 0.006 M NaCl, pH 6.9) to yield final concentrations ranging from 1.9 to 1000 μg/mL. A reaction mixture containing 200 μL of α-amylase solution (2 U/mL) and 200 μL of extract was incubated at 30 °C for 10 min. Subsequently, 200 μL of starch solution (1% *w*/*v* in water) was added and incubated for 3 min. The reaction was terminated by adding 200 μL DNSA reagent (prepared by dissolving 12 g sodium potassium tartrate tetrahydrate in 8 mL of 2 M NaOH and 20 mL of 96 mM DNSA solution) and boiling in a water bath at 85–90 °C for 10 min. After cooling to room temperature, the mixture was diluted with 5 mL of distilled water, and absorbance was measured at 540 nm using a UV-Visible Biosystem 310 spectrophotometer (Biosystem, Barcelona, Spain).

% α amylase inhibition = [(A_blank_ − A_sample_) / A_blank_] × 100

#### 2.2.2. α-Glucosidase-Inhibitory Activity

The α-glucosidase-inhibitory activity of *T. gratilla* alcoholic extract was assessed following the procedure of Pistia Brueggeman and Hollingsworth (2001), with minor modifications [25]. Varying concentrations of the extract (1.97–1000 μg/mL; 50 μL) were mixed with 10 μL of α-glucosidase enzyme solution (1 U/mL, Sigma, St. Louis, MO, USA) and 125 μL of phosphate buffer (0.1 M, pH 6.8). The mixture was incubated at 37 °C for 20 min. Subsequently, the reaction was initiated by adding 20 μL of 1 mM p-nitrophenyl-α-D-glucopyranoside (pNPG) as the substrate, followed by incubation for 30 min. The reaction was terminated by introducing 50 μL of 0.1 N Na_2_CO_3_, and absorbance was recorded at 405 nm using a Biosystem 310 Plus spectrophotometer. The inhibitory activity was calculated using the following equation:

% Inhibition = [(A_blank_ − A_sample_) / A_blank_] × 100

The IC_50_ value, representing the concentration of extract required to inhibit 50% of the enzyme activity, was determined by plotting the percentage inhibition against the extract concentration (1.97–500 μg/mL) and applying regression analysis.

### 2.3. In Vivo Antidiabetic Assay

#### 2.3.1. Preparation of *T. gratilla*-NS

*T. gratilla*-NS was prepared using the nanoprecipitation method [26,27]. Briefly, 2.0 g of alcoholic extract was dissolved in 10 mL of methanol–acetone mixture (3:1 ratio) and then added to 100 mL of water containing 2% *w*/*v* PVP (PVP-K25). The solution was stirred at 1000 rpm using an overhead stirrer (Daihan Scientific Co., Gimpo, Republic of Korea) for 2 h to allow PVP to equilibrate on the nanoparticle surface and to ensure the complete evaporation of organic solvents. The resulting *T. gratilla*-NS samples were then stored in the refrigerator for further examination.

##### Particle Size Analysis

The particle size and polydispersity index (PDI) of the *T. gratilla*-NS were measured using a Zetasizer ZS90 (Malvern Instruments, Malvern, Worcestershire, UK). Before analysis, samples were diluted with double-distilled water, and all measurements were conducted in triplicate.

##### Scanning Electron Microscopy Analysis

The shape and surface morphology of *T. gratilla*-NS were examined using a scanning electron microscope (JSM-5400LV SEM, Jeol, Tokyo, Japan). A drop of the diluted nanosuspension was placed onto carbon-coated copper grids, sputter-coated with gold, and air-dried at room temperature before imaging. The sample was then examined at an acceleration voltage of 15 kV.

#### 2.3.2. In Vivo Model

##### Animals

Healthy adult male Sprague–Dawley rats (200–250 g) were obtained from the Experimental Animal Unit, Faculty of Veterinary Medicine, Zagazig University. The study was conducted with the approval of the Research Ethics Committee (ZU-IACUC) committee in accordance with the U.K. Animals (Scientific Procedures) Act, 1986, and in compliance with the ARRIVE guidelines, Faculty of Veterinary Medicine, Zagazig University (Approval No. ZU-IACUC/2/F/111/2025). For acclimatization, the animals were housed individually for two weeks under standardized laboratory conditions, with the temperature maintained at 25 ± 1 °C, relative humidity at 50–60%, and a 12:12 h light–dark photoperiod in an air-conditioned environment. All rats were provided with standard rodent chow, free access to water, and wood shavings as bedding material.

#### 2.3.3. Experimental Design

##### Induction of Diabetes

After overnight fasting, diabetes was induced by a single intraperitoneal injection of alloxan monohydrate (150 mg/kg). The rats were administered glucose for 48 h to prevent their insulin levels from reaching fatal levels [28]. Following 72 h, the withdrawal of blood samples from the veins of the rats’ tails and their analysis using the glucometer to determine their blood glucose levels were performed. Rats were chosen for the research project based on their fasting blood glucose level being equal to or greater than 250 mg/dL, to confirm their diabetes status.

##### Experimental Groups

Forty rats were divided into four groups, with each group consisting of ten rats selected at random, and the procedure was carried out according to the following method [29]. The first one is the control group, while the second one comprises rats with diabetes. The third group includes diabetic rats given glibenclamide at a dose of 5 mg/kg b.w. Orally for 21 consecutive days. The fourth group consisted of diabetic rats administered a daily oral dose of 200 mg/kg of *T. gratilla*-NS formulation for 21 consecutive days.

The rats were fasted overnight following the last treatment. The rats were anesthetized before obtaining blood samples using cardiac puncture. To extract serum, blood samples were gathered in tubes without an anticoagulant, and the plasma was isolated by using tubes with sodium fluoride. The pancreatic tissues were removed and divided into two sections. One part was homogenized, and then the homogenates were used for antioxidants and oxidative stress biomarkers. The second portion of the sample was used for the histopathological evaluation.

##### Biochemical Analysis

Insulin levels were measured utilizing the rat insulin ELISA method (ERINS, Thermo Fisher Scientific, Waltham, MA, USA), whereas fasting blood glucose levels were assessed using the glucose oxidase technique (Agappe Diagnostics Ltd., Kochi, India). The HOMA-IR value was determined by utilizing the subsequent equation [30]:

[(Fasting glucose (mmol/L)) × fasting insulin (µIU/mL))/22.5)].

HOMA-β was determined by employing the subsequent equation [30]:

[(20 × fasting insulin (µU/mL))/(fasting plasma glucose (mg/dl) − 63)]

##### Determination of Oxidant/Antioxidant Status in Pancreatic Tissue

Malondialdehyde (MDA) concentration in pancreatic tissue was measured according to Ohkawa et al. [31]. Total antioxidant (TAC) levels in pancreatic tissue were estimated according to Koracevic et al. [32]. Superoxide dismutase (SOD) activity in pancreatic tissue was determined according to Melchioretto et al. [33].

##### Histopathological Examination of Pancreatic Tissue

The pancreatic tissue samples from each group were placed in 10% neutral buffered formalin for 48 h for fixation. They were then dehydrated in a series of alcohol concentrations, cleared in xylol, and finally immersed in melted paraffin wax at 59 °C. The paraffin wax blocks were then sliced into sections that were 5 μm thick using a rotary microtome. The paraffin sections that were acquired underwent a process of deparaffinization followed by rehydration in increasing concentrations of alcohol. They were then stained with hematoxylin and eosin (H&E) to enable histopathological assessment [34].

### 2.4. Chemical Profiling of T. gratilla Using GC-Ms

The crude extract of *T. gratilla* was dissolved in an organic solvent and measured according to the following methods [35,36]. The GC-MS system (Agilent, Santa Clara, CA, USA, 7890B GC System, 5977B MSD) was equipped with a DB-5MS column (30 m × 0.25 mm internal diameter, 0.25 μm film thickness). Helium gas was employed as the carrier, and the inlet temperature was 300 C. The analysis was conducted under a gradient temperature program with a 1 µL injection volume. The electron impact ionization was set at 70 eV, covering a mass-to-charge (*m/z*) range of 50–800, with a solvent delay of 7.3 min. Identification of individual constituents was performed by comparing retention times and spectral fragmentation patterns against the Wiley and NIST Mass Spectral Library databases.

### 2.5. Spectroscopic Determination of Total Phenolic Content (TPC)

The total phenolic content of the *alcoholic extract of T. gratilla* was measured using the Folin–Ciocalteu colorimetric method [37,38]. In brief, 1000 µg of the alcoholic extract was dissolved in 2 mL of methanol, and 500 μL of this solution was combined with 2.5 mL of Na_2_Co_3_ and diluted Folin–Ciocalteu reagent (Merck, Darmstadt, Germany). This mixture was subjected to vigorous agitations for seconds and kept at room temperature for 2 h. Finally, the absorbance was measured at 765 nm using a Milton Roy Spectronic 1201 spectrophotometer (Milton Roy, St. Petersburg, FL, USA). A standard calibration curve was prepared using gallic acid solutions (15.62–500 µg/mL), and absorbance values of the extract samples were interpolated from this curve to obtain the equivalent gallic acid concentration (µg/mL). The total phenolic content was then calculated and expressed as milligrams of gallic acid equivalents per gram of dry extract (mg GAE/g extract) using the following equation:

$$\mathrm{TPC}\;(\mathrm{mg}\;\mathrm{GAE}/g\;\mathrm{extract})=\frac{C\times V}{m}$$

where *C* is the concentration obtained from the calibration curve (µg/mL), *V* is the volume of extract (mL), and *m* is the weight of the extract (mg). The experiment was carried out in a triplicate manner.

### 2.6. Statistical Analysis

Statistical analyses were conducted using GraphPad Prism version 8 (GraphPad Software Inc., San Diego, CA, USA). Data are expressed as mean ± standard deviation (SD). Comparisons were assessed by one-way ANOVA, and differences were considered statistically significant at *p* < 0.05.

## 3. Results

### 3.1. In Vitro Antidiabetic Assay

#### 3.1.1. α-Amylase-Inhibitory Activity

The alcoholic extract of the sea urchin*T. gratilla* was tested for its α-amylase-inhibitory activity using a standard in vitro enzyme inhibition assay with acarbose as a positive control. The extract showed significant inhibition of enzyme activity in a dose-dependent manner, as illustrated in Table 1 and Figure S1, with an IC_50_ value of 5.31 ± 0.05 µg/mL compared to acarbose (3.15 ± 0.09 µg/mL) (Table 1).

#### 3.1.2. α-Glucosidase-Inhibitory Activity

As shown in Table 1 and Figure S2, the alcoholic extract of *T. gratilla* was evaluated as an inhibitor for α-glucosidase enzyme with acarbose as a positive control. The extract showed a moderate enzyme inhibition with an IC_50_ of 21.36 ± 0.06 µg/mL, compared to an IC_50_ of 2.21 ± 0.04 µg/mL of acarbose.

Values are mean ± SD (n = 3). IC_50_ = concentration required to inhibit 50% of enzyme activity. Assays were performed in vitro using standard protocols with starch (α-amylase) and p-nitrophenyl-α-D-glucopyranoside (α-glucosidase) as substrates.

### 3.2. In Vivo Antidiabetic Assay

#### 3.2.1. Nanosuspension Formulation of *T. gratilla* (*T. gratilla*-NS)

A nanosuspension of *T. gratilla* was successfully prepared by using the nanoprecipitation method. The formulation containing PVP at 2.0% concentration showed satisfactory zeta potential and particle size. The morphology of *T. gratilla* nanosuspensions revealed almost spherical nanoparticles falling within the nano size range, confirming the nanonization process.

##### Particle Size Distribution

**Figure 1:**
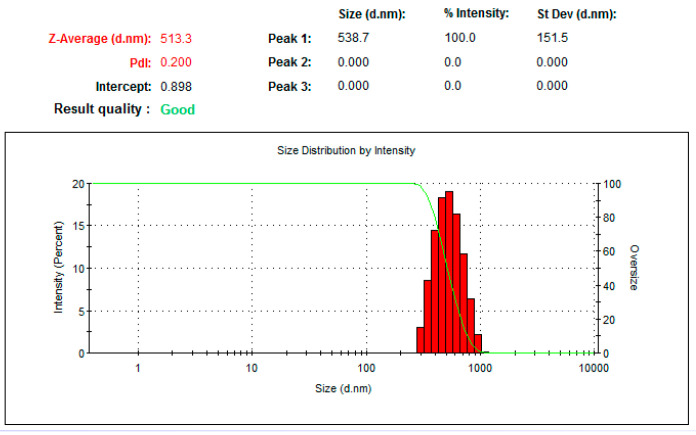
Average particle size distribution and polydispersity index (PDI) of *T. gratilla-NS* as determined by dynamic light scattering (DLS). Data represent the mean of three independent measurements.

Particle size is a key determinant of the physical stability, dissolution rate, and biological efficacy of *T. gratilla*-NS. The formulated *T. gratilla*-NS exhibited a particle size of 513.3 ± 7.4 nm with a PDI of 0.2, indicating a uniform particle distribution (Figure 1).

##### Imaging of *T. gratilla*-NS

**Figure 2:**
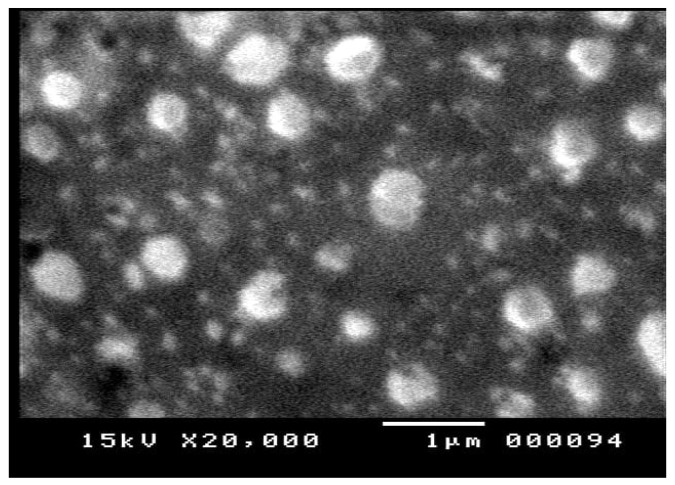
Scanning electron microscopy (SEM) image of *Tripneustes gratilla* nanosuspension showing particle morphology. The image was captured at 20,000× magnification with an accelerating voltage of 15 kV. Scale bar = 1 μm.

The scanning electron microscopy (SEM) image of the *T. gratilla*-NS (Figure 2) revealed nanoparticles within the nanometer scale. The particles appear to be uniformly distributed without noticeable aggregation, consistent with the low PDI value obtained from the Zetasizer analysis. Additionally, the particles exhibited a roughly spherical shape.

#### 3.2.2. In Vivo Antidiabetic Model

##### Impacts of *T. gratilla*-NS on Blood Glucose (BG), Insulin, and HOMA Indices

**Figure 3:**
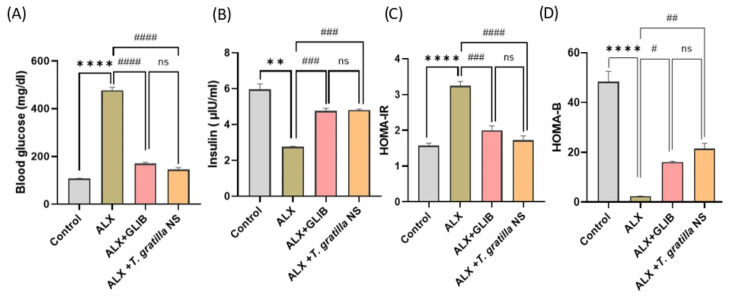
(**A**–**D**) Effects of *T. gratilla*-NS on glucose metabolism and insulin sensitivity in alloxan-induced diabetic rats. (**A**) Blood glucose (mg/dL), (**B**) insulin (µIU/mL), (**C**) HOMA-IR (Homeostatic Model Assessment—Insulin Resistance), and (**D**) HOMA-B (β-cell function). Experimental groups: Control (untreated), ALX (alloxan-induced diabetic rats), ALX + GLIB (alloxan-induced diabetic rats treated with glibenclamide, 5 mg/kg), and ALX + *T. gratilla*-NS (200 mg/kg). Data are presented as mean ± SD (n = 10). ** *p* < 0.01, **** *p* < 0.0001 vs. control; # *p* < 0.05, ## *p* < 0.01, ### *p* < 0.001, #### *p* < 0.0001 vs. ALX.; ns = not significant.

Compared to the control group, alloxan-induced diabetic rats showed a significant elevation in blood glucose levels (3.5-fold) and Homeostatic Model Assessment—Insulin Resistance (HOMA-IR) (1.2-fold), along with a significant reduction in insulin (0.5-fold) and HOMA-B (0.9-fold) compared to healthy animals (*p* < 0.01). However, rats treated with *T. gratilla*-NS showed a considerable drop in blood glucose levels by 0.7 fold and a marked decrease in HOMA-IR by 0.5 fold, respectively, in comparison to the diabetic group. Furthermore, *T. gratilla*-NS-treated groups exhibited a substantial increase in insulin by 0.8 fold and HOMA-B by 9 fold, compared to the alloxan–diabetic group. Interestingly, no statistically significant differences in the above indices were observed between the positive control group and diabetic rats given *T. gratilla*-NS (Figure 3A–D).

##### Impacts of *T. gratilla*-NS on the Oxidant/Antioxidant Parameters

**Figure 4:**
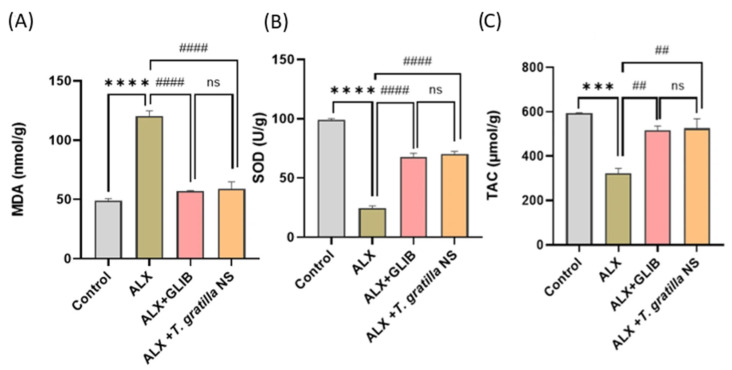
(**A**–**C**) Effects of *Tripneustes gratilla* nanosuspension (NS) on oxidative stress and antioxidant parameters in alloxan-induced diabetic rats. (**A**) Malondialdehyde (MDA, nmol/g), (**B**) superoxide dismutase (SOD, U/g), and (**C**) total antioxidant capacity (TAC, µmol/g). Experimental groups: Control, ALX (alloxan-induced diabetic rats), ALX + GLIB (alloxan-induced diabetic rats treated with glibenclamide, 5 mg/kg), and ALX + *T. gratilla*-NS (200 mg/kg). Data are presented as mean ± SD (n = 10). *** *p* < 0.001, **** *p* < 0.0001 vs. control; ## *p* < 0.01, #### *p* < 0.0001 vs. ALX; ns = not significant.

Alloxan-treated rats showed a notable increase in levels of the lipid peroxidation marker Malondialdehyde (MDA) in pancreatic tissues by 1.5 fold and a decrease in levels of the antioxidant marker Superoxide Dismutase (SOD) and Total Antioxidant Capacity (TAC) by 0.8 fold and 0.5 fold, respectively, in comparison to the healthy diabetic control group. Conversely, the oral intake of *T. gratilla*-NS led to a considerable decrease in MDA concentration by 0.5 fold and an increase in SOD and TAC levels by 2 fold and 0.8 fold, respectively, compared to the alloxan–diabetic group, with results closely comparable to the positive-control-treated groups (Figure 4A–C).

##### Histopathological Examination

**Figure 5:**
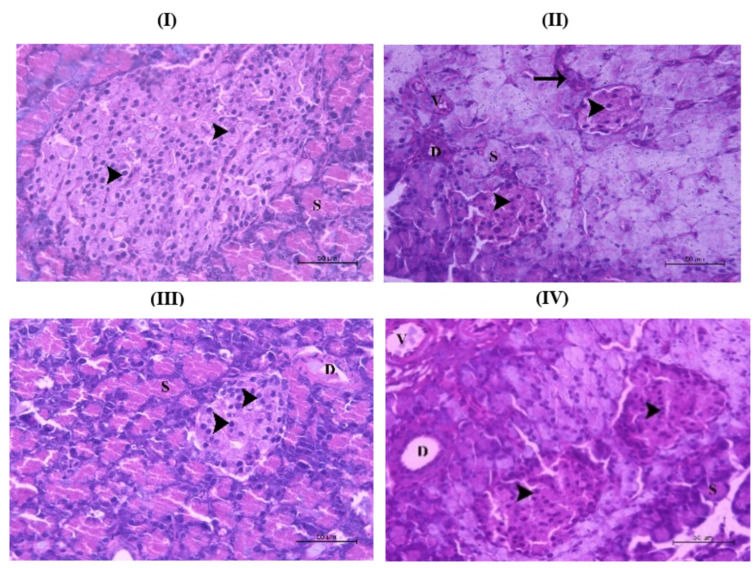
(**I**) Control (non-treated) group showed normal pancreatic tissue architecture with normal pancreatic acini (S) and normal pancreatic islets containing normal cells (arrowhead). (**II**) ALX (alloxan-induced diabetic group) showed massive disorientation of pancreatic tissue in the form of inflammatory changes (arrow), fibrosis around the pancreatic duct (D), and congestion of pancreatic vessels (V), as well as marked reduction in size of the islets. The pancreatic islets’ cells showed a reduction in cell number with severe degeneration (arrowhead). (**III**) ALX+ GLIB (alloxan-induced diabetic rats receiving glibenclamide as a positive control) showed pancreatic tissue that appeared more or less normal with normal pancreatic acini (S) and pancreatic ducts (D). The islets appeared smaller in size with normal cells (arrowhead). (**IV**) (ALX + *T. gratilla*-NS) showed normal pancreatic tissue with normal pancreatic acini (S), pancreatic ducts (D), and pancreatic vessels (V). The islets appeared larger in size with normal cells (arrowhead) (H&E Stain X400).

To assess the protective effects of the treatments on pancreatic integrity, histological examination of pancreatic sections was performed at the end of the experiment for all study groups (Figure 5I–IV). Sections from the healthy control (untreated) group exhibited normal pancreatic histology with intact acini (S) and ducts (D). In contrast, investigation of sections from the alloxan-induced group showed pathological changes in the form of severe structural disruption, characterized by inflammatory cell infiltration, periductal fibrosis, vascular congestion, and a pronounced shrinkage of the islets. Within the ilets, a marked decline in cell number and extensive degeneration was observed, as well (arrowhead). The positive control group (ALX + GLIB), receiving glibenclamide, showed pancreatic tissue largely comparable to the normal group, with intact acini (S) and ducts (D). However, the islets appeared relatively smaller, though containing morphologically normal cells (arrowhead). The *T. gratilla*-treated group, which received oral dose of nanosuspension formulation (ALX + *T. gratilla*-NS) showed visible improvement in the pancreatic morphology with enlarged islets containing normal pancreatic cells.

### 3.3. Chemical Characterization of T. gratilla Using GC-MS

**Figure 6:**
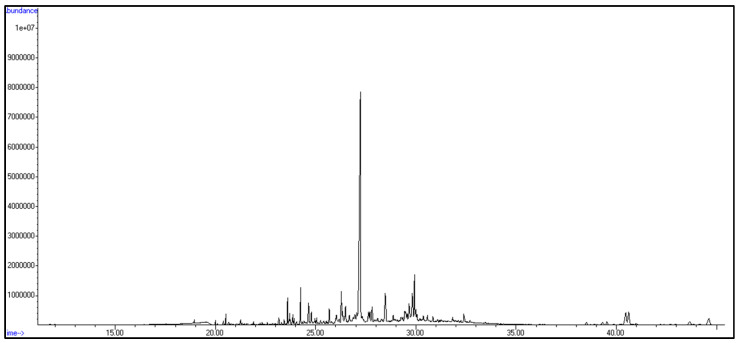
GC-MS chromatogram of the alcoholic extract of the sea urchin *T. gratilla.*

Gas chromatography–mass spectrometry analysis of the alcoholic extract of *T. gratilla* revealed several metabolites belonging to diverse chemical classes of natural products, including terpenoids, steroids, lipid derivatives, and nitrogenous compounds, which include amino acids and other related derivatives (Table S1, Figure 6 and Figures S3–S8). Compound identification was achieved by analyzing retention times, mass spectra, and fragmentation patterns, which were then compared to standard reference data from the Wiley and NIST libraries.

Terpenes constituted the major class of metabolites identified in the alcoholic extract of *T. gratilla* via GC-MS analysis and were represented by fifteen compounds, including nine sesquiterpenes, five diterpenes, and one monoterpene. The most intense peak was assigned to germacrene (Rt = 27.22 min). The sesquiterpenes included two bicyclic sesquiterpenes, (−)-β-caryophyllene epoxide (Rt = 29.35 min) and caryophylla-3,8(13)-dien-5*β*-ol (Rt = 31.06 min); one aromatic sesquiterpene, L-calamenene (Rt = 21.34 min); a spiro type sesquiterpene, chamigrene (Rt = 26.36 min); two acyclic sesquiterpenes, nerolidol (Rt = 23.71 min) and β-farnesene (Rt = 20.00 min); one monocyclic derivative, α-bisabolene epoxide (Rt = 30.21 min); and vulgarol A (Rt = 31.18 min). The detected diterpenes were mainly cembranoid derivatives, including cembra-4,7,11,15-tetraen-3-ol(Rt = 27.32 min), (R)-(−)-cembrene (Rt = 27.92 min), and 3,4-epoxycembra-7,11,15-triene (Rt = 29.82 min). The remaining two diterpens detected were geranylgeraniol (Rt = 28.48 min) and thunbergol (Rt = 30.85 min), while the one identified monoterpene was loliolide (Rt = 22.98 min).

Additionally, five steroids were identified, including cholesterol (Rt = 38.51 min), ergosta-5,24-dien-3β-ol (Rt = 40.47 min), 22,23-dibromostigmasterol acetate (Rt = 36.31 min), androstane-3,17-dione (Rt = 25.40 min), and gorgosterol (Rt = 46.48 min).

The lipid derivatives included three polyunsaturated fatty acids: 2,5,10-undecatrienoic acid methyl ester (Rt = 30.58 min), 5,8,11,14,17-eicosapentaenoic acid methyl ester (Rt = 27.81 min), and arachidonic acid (eicosa-5,8,11,14-tetraenoic acid) (Rt = 27.62 min); two monounsaturated fatty acids: 11-hexadecenoic acid (Rt = 24.24 min) and methyl palmitoleate (Rt = 24.05 min); and four saturated fatty acid esters: methyl palmitate (Rt = 27.81 min), methyl stearate (Rt = 26.68 min), tetradecyl octadecanoate (Rt = 23.28 min), and methyl tetradecanoate (Rt = 22.59 min). The one identified amino acid was methionine (Rt = 18.93 min), together with one purine derivative and two alkaloidal compounds.

### 3.4. Total Phenolic Content of the Alcoholic Extract of T. gratilla

The total phenolic content (TPC) of the alcoholic extract of *T. gratilla* was determined to be 54.26 ± 1.27 mg GAE g^−1^ extract, from the gallic acid calibration curve (Figure S9). The calibration curve was constructed to obtain the equivalent gallic acid concentration. The resulting concentration was then converted to a mg GAE g^−1^ extract according to the method described by Singleton and Rossi (1965) [37].

## 4. Discussion

Globally, diabetes mellitus is considered one of the major public health concerns based on its long-term complications and economic challenges for individuals, families, and governments. The disease is associated with relative insulin deficiency and diminished cellular response to insulin. Postprandial hyperglycemia can aggravate diabetic complications, and after a meal, the two enzymes responsible for the digestion of complex carbohydrates are α-amylase and α-glucosidase [1,2,3,4,5,6].

In this research, alcoholic extract of the sea urchin *T. gratilla* showed potent amylase-inhibitory activity and moderate activity toward the α-glucosidase enzyme. This activity may be attributed to the synergy between metabolites that exist in the sea urchin and are detected using GC-MS. Oxygenated sesquiterpenes such as β-caryophyllene epoxide, calamenene, nerolidol, and bisabolene epoxide can interact and fit into hydrophobic moieties via van der Waals forces with carbohydrate-degrading enzymes. Furthermore, intrinsically biosynthesized phenolic compounds, notably polyhydroxylated naphthoquinones (PHNQ) like the echinochromes, alongside dietary phenolics derived from brown algae, may also contribute significantly to the observed inhibition of α-amylase and α-glucosidase [39,40].

Additionally, diterpenes like cembrene derivatives, thunbergol, and geranylgeraniol, can interact and inhibit mainly α-glucosidase enzyme, and steroids with ergostane nucleus and PUFAs may also modulate carbohydrate metabolism and enhance enzyme-inhibitory activity [41,42]. These suggestions are consistent with previous reports, which illustrate the activity of these classes of metabolites and the antidiabetic potential of the Echinoidea extract *Echinometra mathaei* [43]. The above results illustrate the activity of the alcoholic extract of *T. gratilla.* Among the main problems associated with in vivo experiments using either marine or plant sources were the low water solubility and stability, which led to poor systemic absorption and reduced therapeutic efficacy [22,23]. Consequently, a nanosuspension was prepared from the alcoholic extract of *T.gratilla* to enhance its pharmacological properties. This approach aimed to increase the dissolution rate and oral bioavailability, thereby facilitating greater metabolite delivery to target tissues and enhancing overall therapeutic potency [22,23,44]. The *T. gratilla*-NS was tested via an in vivo antidiabetic assay, and diabetes was induced using alloxan. In the three groups treated with alloxan, alloxan led to elevated blood glucose and reduced serum insulin, resulting in increased HOMA-IR and reduced HOMA-*β* values [45]. These effects are mainly due to two mechanisms: inhibition of glucose-induced insulin secretion and induction of insulin-dependent diabetes via excessive ROS generation, causing *β*-cell necrosis [46].

Diabetes is further characterized by diminished insulin release from dysfunctional *β*-cells under hyperglycemia. The HOMA model is a standard tool to evaluate insulin resistance and *β*-cell activity based on fasting glucose and insulin [47]. In agreement with previous findings, diabetic rats displayed higher HOMA-IR and lower HOMA-*β* than controls, confirming *β*-cell impairment. The HOMA model is recognized as an indicator of insulin resistance and pancreatic *β*-cell activity, as it is calculated from fasting glucose and insulin values [47,48]. In this study, diabetic rats (group 2) exhibited a significant increase in HOMA-IR alongside a reduction in HOMA-*β* when compared with controls. These alterations reflect impaired *β*-cell function and enhanced insulin resistance, in agreement with previous findings [47,48].

Our findings demonstrated that administration of *T. gratilla*-NS significantly caused a considerable drop in blood glucose and HOMA-IR values while markedly increasing insulin levels and HOMA-*β* compared with the diabetic group. The outcomes were closely comparable to those observed in the glibenclamide-treated rats.

The antidiabetic potential of *T. gratilla*-NS may be attributed to its content of terpenes, steroids, and polyunsaturated fatty acids (PUFAs), which collectively enhance insulin sensitivity, decrease blood glucose level, and protect *β*-cells [49]. *β*-Caryophyllene has been shown to improve insulin release and regulate carbohydrate-metabolizing enzymes in a dose-dependent fashion in diabetic models [50]. Additionally, previous research reported that β-caryophyllene is a selective full agonist of the cannabinoid receptor-2 (CB2R), whose activation promotes insulin secretion from pancreatic β-cells [51]. Similarly, stigmasterol demonstrated antidiabetic activity in animal studies by lowering blood glucose, urea, and creatinine, while stimulating insulin uptake and exerting antihyperglycemic effects [52]. Moreover, in PUFAs such as eicosapentaenoic acid and undecatrienoic acid, methyl ester improves membrane fluidity and increases insulin receptor density and affinity, thereby enhancing insulin action and reducing the risk of insulin resistance and the development of T2DM [49].

One of the key mechanisms behind diabetes and its complications is the excessive generation of reactive oxygen species (ROS), which promotes lipid peroxidation while diminishing antioxidant enzyme activity and glutathione (GSH) levels [53]. In diabetic rats, persistent hyperglycemia elevates ROS within pancreatic tissue, leading to β-cell damage and eventual dysfunction [53]. In this study, all hallmarks of oxidative stress were observed in alloxan-induced diabetic rats with higher MDA concentrations in the pancreas, reflecting lipid peroxidation and reduced antioxidant markers such as SOD and TAC.

Oral administration of *T. gratilla*-NS significantly decreased pancreatic MDA and elevated SOD and total antioxidant capacity (TAC) compared with the diabetic group. Similarly, treatment with GLIB restored antioxidant balance by enhancing SOD and TAC, while lowering MDA levels, confirming earlier reports of its antioxidant capacity [54]. The in vivo antioxidant activity of *T. gratilla*-NS might be linked to synergistically detected bioactive compounds, including β-caryophyllene, L-calamenene, nerolidol, geranylgeraniol, loliolide, eicosapentaenoic acid, and D-methionine [55]. Phenolic derivatives existed in significant amounts in *T. gratilla* compared to other sea urchin species. In particular, the methanolic extract of *Stomopneustes variolaris* gonads contained approximately 9.90 ± 0.01 mg GAE/g, whereas the coelomic fluid and red blood cells of the black sea urchin *Arbacia lixula* exhibited even higher phenolic concentrations, reaching 15 ± 2.4 mg GAE/g. In contrast, the shell of *Echinometra mathaei* displayed a markedly lower total phenolic content of 0.1781 ± 0.017 mg GAE/g of extract. The elevated phenolic concentration in *T. gratilla* (54.26± 1.27 mg. GAE g^−1^ of extract) likely contributes to antioxidant capacity and participates in the overall antidiabetic potential [56,57,58,59,60,61].

These results suggest that *T. gratilla*-NS may slow carbohydrate digestion and consequently avoid sudden spikes in blood glucose levels. In parallel, *T. gratilla* decreased ROS, increased insulin levels, and improved tissue sensitivity to it.

The current study is limited by the absence of clinical data, and the lack of mechanistic exploration at the molecular level. Future investigations should employ bio-guided isolation of bioactive metabolites, molecular pathway elucidation, and long-term preclinical and clinical evaluations to justify the antidiabetic potential of the *T. gratilla* nanosuspension.

## 5. Conclusions

The nanosuspension of *T. gratilla* alcoholic extract exhibited significant antidiabetic efficacy by enhancing insulin secretion, improving glucose regulation, and restoring oxidative balance in diabetic rats. These effects were supported by significant inhibition of α-amylase and moderate suppression of α-glucosidase, indicating dual action in controlling both fasting and postprandial hyperglycemia. The presence of bioactive compounds such as sesquiterpenes, steroids, and polyunsaturated fatty acids and phenolics likely contributes to this effect through antioxidant, insulin-sensitizing, and enzyme-modulating mechanisms. Additionally, the nanosuspension form improved the bioavailability and metabolic stability of these constituents, enhancing their pharmacological response. Overall, *T. gratilla*-NS represents a promising natural candidate for diabetes management, warranting further bio-guided isolation of its active antidiabetic metabolites, along with detailed mechanistic, pharmacokinetic, and clinical investigations to validate its therapeutic efficacy and safety profile.

## Figures and Tables

**Table 1:** α-Amylase- and α-glucosidase-inhibitory activities of the alcoholic extract of *T. gratilla.*

Sample	In Vitro Antidiabetic Assay	
	α-Amylase IC_50_ (µg/mL)	α-Glucosidase IC_50_ (µg/mL)
Alc. Ext of *T. gratilla*	5.31 ± 0.004	21.36 ± 0.06
Positive control	3.15 ± 0.007	2.21 ± 0.04

## Data Availability

All data supporting the findings of this study are available in the Supplementary Materials associated with this article.

## Supplementary Materials

The following supporting information can be downloaded at: https://www.mdpi.com/article/10.3390/cimb48010008/s1.

## Abbreviations

The following abbreviations are used in this manuscript:

T1DM	Type 1 insulin-dependent diabetes mellitus
T2DM	Type 2 insulin-dependent diabetes mellitus
ALX	Alloxan
HOMA	Homeostatic Model Assessment
HOMA-IR	Homeostatic Model Assessment—Insulin Resistance
NS	Nanosuspension
MDA	Malondialdehyde
ROS	Reactive Oxygen Species
SOD	Superoxide Dismutase
TAC	Total Antioxidant Capacity
GLIB	Glibenclamide
PVP	Polyvinylpyrrolidone

## References

[1] Deepthi B., Sowjanya K., Lidiya B., Bhargavi R.S., Babu P.S. (2017). A modern review of diabetes mellitus: An annihilatory metabolic disorder. Silico Vitro Pharmacol..

[2] Weber C. (2010). Challenges in funding diabetes care: A health economic perspective. Expert Rev. Pharmacoecon. Outcomes Res..

[3] Nedosugova L.V., Markina Y.V., Bochkareva L.A., Kuzina I.A., Petunina N.A., Yudina I.Y., Kirichenko T.V. (2022). Inflammatory mechanisms of diabetes and its vascular complications. Biomedicines.

[4] Elhefnawy M.E., Ghadzi S.M.S., Noor Harun S. (2022). Predictors associated with type 2 diabetes mellitus complications over time: A literature review. J. Vasc. Dis..

[5] Goyal Y., Verma A.K., Bhatt D., Rahmani A.H., Dev K. (2020). Diabetes: Perspective and challenges in modern era. Gene Rep..

[6] El-Kebbi I.M., Bidikian N.H., Hneiny L., Nasrallah M.P. (2021). Epidemiology of type 2 diabetes in the Middle East and North Africa: Challenges and call for action. World J. Diabetes.

[7] Stein S.A., Lamos E.M., Davis S.N. (2013). A review of the efficacy and safety of oral antidiabetic drugs. Expert Opin. Drug Saf..

[8] Singh S., Bhat J., Wang P.H. (2013). Cardiovascular effects of anti-diabetic medications in type 2 diabetes mellitus. Curr. Cardiol. Rep..

[9] Jia W., Gao W., Tang L. (2003). Antidiabetic herbal drugs officially approved in China. Phytother. Res..

[10] Blahova J., Martiniakova M., Babikova M., Kovacova V., Mondockova V., Omelka R. (2021). Pharmaceutical drugs and natural therapeutic products for the treatment of type 2 diabetes mellitus. Pharmaceuticals.

[11] Casertano M., Vito A., Aiello A., Imperatore C., Menna M. (2023). Natural bioactive compounds from marine invertebrates that modulate key targets implicated in the onset of type 2 diabetes mellitus (T2DM) and its complications. Pharmaceutics.

[12] Sibiya A., Jeyavani J., Sivakamavalli J., Ravi C., Divya M., Vaseeharan B. (2021). Bioactive compounds from various types of sea urchin and their therapeutic effects—A review. Reg. Stud. Mar. Sci..

[13] Pearse J.S. (2006). Ecological role of purple sea urchins. Science.

[14] Li C., Haug T., Moe M.K., Styrvold O.B., Stensvåg K. (2010). Centrocins: Isolation and characterization of novel dimeric antimicrobial peptides from the green sea urchin, *Strongylocentrotus droebachiensis*. Dev. Comp. Immunol..

[15] Mishchenko N.P., Krylova N.V., Iunikhina O.V., Vasileva E.A., Likhatskaya G.N., Pislyagin E.A., Fedoreyev S.A. (2020). Antiviral potential of sea urchin aminated spinochromes against herpes simplex virus type 1. Mar. Drugs.

[16] Abdelkarem F.M., Desoky E.E.K., Nafady A.M., Allam A.E., Mahdy A., Ashour A., Shimizu K. (2022). *Diadema setosum*: Isolation of bioactive secondary metabolites with cytotoxic activity toward human cervical cancer. Nat. Prod. Res..

[17] AbouElmaaty E.E., Ghobashy A.A., Hanafy M.H., Yassien M.H., Ahmed M.I., Hamed M.M. (2020). Preliminary bioassay on antibacterial effects of *Tripneustes gratilla* extracts from the Red Sea, Egypt. Egypt. J. Aquat. Biol. Fish..

[18] Abdelaziz Y.A., Khallaf I.S., Alian A., Ibrahim A.A., Desoky E.E.K., Abdelkarem F.M. (2024). LC–MS-based chemical profiling of Aristotle’s lantern and viscera of the sea urchin *Echinometra mathaei* collected from the Red Sea and evaluation of their antiviral activity. Future J. Pharm. Sci..

[19] Chen Y.C., Chen T.Y., Chiou T.K., Hwang D.F. (2013). Seasonal variation on general composition, free amino acids and fatty acids in the gonad of Taiwan’s sea urchin *Tripneustes gratilla*. J. Mar. Sci. Technol..

[20] Chen Y.C., Hwang D.F. (2014). Evaluation of antioxidant properties and biofunctions of polar, nonpolar, and water-soluble fractions extracted from gonad and body wall of the sea urchin *Tripneustes gratilla*. Fish. Sci..

[21] Das S., Sharangi A.B. (2020). Nanotechnology: A potential tool in exploring herbal benefits. Functional Bionanomaterials: From Biomolecules to Nanoparticles.

[22] Ma R., Xiang L., Zhao X., Yin J. (2022). Progress in preparation of sea urchin-like micro-/nanoparticles. Materials.

[23] Teixeira L.M., Reis C.P., Pacheco R. (2025). Marine-derived compounds combined with nanoparticles: A focus on the biomedical and pharmaceutical sector. Mar. Drugs.

[24] Wickramaratne M.N., Punchihewa J.C., Wickramaratne D.B.M. (2016). In-vitro alpha amylase inhibitory activity of the leaf extracts of *Adenanthera pavonina*. BMC Complement. Altern. Med..

[25] Pistia-Brueggeman G., Hollingsworth R.I. (2001). A preparation and screening strategy for glycosidase inhibitors. Tetrahedron.

[26] Raza A., Ali T., Naeem M., Asim M., Hussain F., Li Z., Nasir A. (2024). Biochemical characterization of bioinspired nanosuspensions from *Swertia chirayita* extract and their therapeutic effects through nanotechnology approach. PLoS ONE.

[27] Chavda V.P., Vaghela D.A., Solanki H.K., Balar P.C., Modi S., Gogoi N.R. (2025). Nanosuspensions: A new era of targeted therapeutics. J. Drug Deliv. Sci. Technol..

[28] Đurašević S., Nikolić G., Zaletel I., Grigorov I., Memon L., Mitić-Ćulafić D., Vujović P., Đorđević J., Todorović Z. (2020). Distinct effects of virgin coconut oil supplementation on the glucose and lipid homeostasis in non-diabetic and alloxan-induced diabetic rats. J. Funct. Foods.

[29] Shah N.A., Khan M.R. (2014). Antidiabetic effect of *Sida cordata* in alloxan induced diabetic rats. Biomed. Res. Int..

[30] Matthews D.R., Hosker J.P., Rudenski A.S., Naylor B.A., Treacher D.F., Turner R.C. (1985). Homeostasis model assessment: Insulin resistance and beta-cell function from fasting plasma glucose and insulin concentrations in man. Diabetologia.

[31] Ohkawa H., Ohishi N., Yagi K. (1979). Assay for lipid peroxides in animal tissues by thiobarbituric acid reaction. Anal. Biochem..

[32] Koracevic D., Koracevic G., Djordjevic V., Andrejevic S., Cosic V. (2001). Method for the measurement of antioxidant activity in human fluids. J. Clin. Pathol..

[33] Melchioretto E.F., Zeni M., Veronez D.A.L., Neto F.F., Digner I.S., de Fraga R. (2020). Stereological study and analysis of oxidative stress during renal aging in rats. Acta Cir. Bras..

[34] Bancroft J.D., Layton C., Suvarna S.K., Layton C., Bancroft J.D. (2013). The hematoxylin and eosin. Theory and Practice of Histological Techniques.

[35] Hassan M., Bala S.Z., Bashir M., Waziri P.M., Adam R.M., Umar M.A., Kini P. (2022). LC-MS and GC-MS profiling of different fractions of *Ficus platyphylla* stem bark ethanolic extract. J. Anal. Methods Chem..

[36] Aljohani A.K., Maghrabi N.A., Alrehili O.M., Alharbi A.S., Alsihli R.S., Alharthe A.M., Hussein M.F. (2025). Ajwa date extract (*Phoenix dactylifera* L.): Phytochemical analysis, antiviral activity against herpes simplex virus-I and coxsackie B4 virus, and in silico study. Saudi Med. J..

[37] Singleton V.L., Rossi J.A. (1965). Colorimetry of Total Phenolics with Phosphomolybdic–Phosphotungstic Acid Reagents. Am. J. Enol. Vitic..

[38] Marinova D., Ribarova F., Atanassova M. (2005). Total Phenolics and Total Flavonoids in Bulgarian Fruits and Vegetables. J. Univ. Chem. Technol. Metall..

[39] Khamis A.A., Elkeiy M.M., El-Gamal M.M., Saad-Allah K.M., Salem M.M. (2025). Biological and Molecular Efficiency of *Paracentrotus lividus* Shell in vitro Study: Antioxidant and Angiogenesis Effects Against T47D Breast Cancer Cell Line Via Nrf2/HMOX-1/and HIF-1α/VEGF Signaling Pathways. Cell Biochem. Biophys..

[40] Mahnashi M.H., Alqahtani Y.S., Alyami B.A., Alqarni A.O., Ayaz M., Ghufran M., Murthy H.A. (2022). Phytochemical Analysis, α-Glucosidase and Amylase Inhibitory, and Molecular Docking Studies on *Persicaria hydropiper* L. Leaves Essential Oils. Evid. Based Complement. Alternat. Med..

[41] Shi Q., Yu S., Zhou M., Wang P., Li W., Jin X., Meng X. (2025). Diterpenoids of Marine Organisms: Isolation, Structures, and Bioactivities. Mar. Drugs.

[42] Lauritano C., Ianora A. (2016). Marine organisms with anti-diabetes properties. Mar. Drugs.

[43] Soleimani S., Moein S., Yousefzadi M., Amrollahi Bioki N. (2021). Antidiabetic and antioxidant properties of sea urchin Echinometra mathaei from the Persian Gulf. J. Kerman Univ. Med. Sci..

[44] Ma Y., Cong Z., Gao P., Wang Y. (2023). Nanosuspensions technology as a master key for nature products drug delivery and in vivo fate. Eur. J. Pharm. Sci..

[45] Ibrahim R.M., Abdelhafez H.M., EL-Shamy S.A.E.M., Eid F.A., Mashaal A. (2023). Arabic gum ameliorates systemic modulation in alloxan monohydrate-induced diabetic rats. Sci. Rep..

[46] Lenzen S. (2008). The mechanisms of alloxan- and streptozotocin-induced diabetes. Diabetologia.

[47] Wallace T.M., Levy J.C., Matthews D.R. (2004). Use and abuse of HOMA modeling. Diabetes Care.

[48] Ebokaiwe A.P., Okori S., Nwankwo J.O., Ejike C.E.C.C., Osawe S.O. (2021). Selenium nanoparticles and metformin ameliorate streptozotocin-instigated brain oxidative-inflammatory stress and neurobehavioral alterations in rats. Naunyn Schmiedebergs Arch. Pharmacol..

[49] Pinel A., Morio-Liondore B., Capel F. (2014). n−3 Polyunsaturated Fatty Acids Modulate Metabolism of Insulin-Sensitive Tissues: Implication for the Prevention of Type 2 Diabetes. J. Physiol. Biochem..

[50] Basha R.H., Sankaranarayanan C. (2014). β-Caryophyllene, a natural sesquiterpene, modulates carbohydrate metabolism in streptozotocin-induced diabetic rats. Acta Histochem..

[51] Gertsch J., Leonti M., Raduner S., Racz I., Chen J.Z., Xie X.Q., Altmann K.H., Karsak M., Zimmer A. (2008). Beta-caryophyllene is a dietary cannabinoid. Proc. Natl. Acad. Sci. USA.

[52] Nualkaew S., Padee P., Talubmook C. (2015). Hypoglycemic activity in diabetic rats of stigmasterol and sitosterol-3-O-β-d-glucopyranoside isolated from *Pseuderanthemum palatiferum* (Nees) Radlk. leaf extract. J. Med. Plants Res..

[53] Yaghmaie P., Heydarian E., Poorbahman N. (2011). The regenerative effects of *Thymus vulgaris* extract on beta cells of pancreas of streptozotocin induced diabetic Wistar rats. Med. Sci. J. Islam. Azad Univ. Tehran Med. Branch.

[54] Gerber P.A., Rutter G.A. (2017). The role of oxidative stress and hypoxia in pancreatic beta-cell dysfunction in diabetes mellitus. Antioxid. Redox Signal..

[55] Khan A.L., Khan H., Hussain J., Adnan M., Hussain I., Khan T., Khan A.R. (2008). Sesquiterpenes: The potent antioxidants. Pak. J. Sci. Ind. Res..

[56] Archana A., Babu K.R. (2016). Nutrient composition and antioxidant activity of gonads of sea urchin *Stomopneustes variolaris*. Food Chem..

[57] Quarta S., Scoditti E., Zonno V., Siculella L., Damiano F., Carluccio M.A., Pagliara P. (2023). In vitro anti-inflammatory and vasculoprotective effects of red cell extract from the Black Sea Urchin Arbacia lixula. Nutrients.

[58] Soleimani S., Moein S., Yousefzadi M., Bioki N.A. (2016). Determination of in vitro antioxidant properties, anti-inflammatory effects, and α-amylase inhibition of purple sea urchin extract of *Echinometra mathaei* from the Persian Gulf. Jundishapur J. Nat. Pharm. Prod..

[59] Pagels F., Garrido I., Teixeira C., Tavares T.G., Costas B., Malcata F.X., Guedes A.C. (2023). Sea urchin (*Paracentrotus lividus*) gut biomass as a co-product with antioxidant and antibacterial potential to supplement aquafeeds. Aquat. Living Resour..

[60] Fedoreyev S.A., Krylova N.V., Mishchenko N.P., Vasileva E.A., Pislyagin E.A., Iunikhina O.V., Lavrov V.F., Svitich Y.V., Ebralidze L.K., Leonova G.N. (2018). Antiviral and antioxidant properties of echinochrome A. Mar. Drugs.

[61] Shah M.A., Khalil A.A., ul Haq I., Khan M.N. (2020). Pharmacological activities of sea urchin bioactive secondary metabolites. Pak. J. Pharm. Sci..

