# Supplementary material for: Antidiabetic Potential of Sea Urchin Tripneustes gratilla Nanosuspension Based on In Vitro Enzyme Inhibition, In Vivo Evaluation, and Chemical Profiling Approaches
Source: Curr Issues Mol Biol. 2025 Dec 21;48(1):8. doi: 10.3390/cimb48010008 (PMC12839668; doi:10.3390/cimb48010008)
Supplement: Supplementary file 1 [file cimb-48-00008-s001.zip › cimb-3901876-supplementary.pdf]

Antidiabetic Potential of Sea urchin *Tripneustes gratilla* Nanosuspension Based on In Vitro Enzyme Inhibition, In Vivo Evaluation, and Chemical Profiling Approaches

Abstract:

Diabetes mellitus represents one of the main health challenges worldwide, characterized by hyperglycemia and long-term serious microvascular and macrovascular complications. Marine organisms are a promising reservoir of bioactive metabolites for developing effective antidiabetic therapies with fewer side effects. The sea urchin *Tripneustes gratilla* (*T. gratilla*) is widely distributed in the Red Sea, with limited reports of its pharmacological activities and chemical characterization. In this study, a nanosuspension formulation of *T. gratilla* extract (*T. gratilla*-NS) was developed to enhance the bioavailability of its bioactive constituents. This study investigated the antidiabetic potential of *T. gratilla* extract through an integrated approach encompassing chemical profiling of the extract, assessment of its alcoholic extract for in vitro inhibitory effects on α-amylase and α-glucosidase, and in vivo evaluation of *T. gratilla*-NS in an alloxan-induced diabetic rat model. We found that the alcoholic extract showed potent inhibitory action toward α-amylase with IC_{50} 5.31 ± 0.05 μg/mL and moderate inhibitory activity toward α-glucosidase with IC_{50} 21.36 ± 0.06 μg/mL. *T. gratilla*-NS significantly increased insulin levels, reduced blood glucose levels, and restored pancreatic damage. Furthermore, it enhanced the levels of superoxide dismutase and total antioxidant capacity with a concomitant decrease in malondialdehyde concentration in pancreatic tissue. The observed activities could be attributed to a wide array of diverse compounds, including terpenes, mainly sesquiterpenes, diterpenes, steroids, and polyunsaturated fatty acids detected by GC-MS, compounds with a phenolic nucleus equal to 54.26± 1.27 mg. GAE/g. This research highlights the dual role of *T. gratilla*-NS in combating diabetes and subsequently attenuating its associated complications.

Keywords: Sea Urchin; antioxidant; α-amylase; α-glucosidase; antidiabetic.

Table S1. List of bioactive compounds identified from the sea urchin *Tripneustes gratilla* extract using gas chromatography–mass spectrometry (GC–MS).

No.	Compounds	Rt. (min.)	Area (%)	Major Fragments (m/z)
	**Terpenoids**			
1	β-Caryophyllene epoxide	29.35	1.21	205, 93, 79, 41
2	Caryophylla-3,8(13)-dien-5β.-ol	28.27	1.01	202, 187, 123, 107
3	α-bisabolene epoxide	30.21	1.09	159, 135, 109, 93
4	Vulgarol A	31.18	0.45	205, 177, 149, 135
5	Germacrene	27.22	37.94	189, 147, 107, 68
6	Chamigrene	26.33	2.04	189, 119, 93, 41
7	L-calamenene	21.33	0.21	159, 144, 129, 105
8	β-Farnesene	20.1	0.12	161, 133, 93, 69
9	Nerolidol	23.71	1.23	204, 161, 136, 69
10	(R)-(-)-Cembrene	27.92	0.61	257, 229, 119, 93
11	3,4-Epoxycembra- 7,11,15-triene	29.82	8.92	273, 255, 216, 93
12	Cembra-4,7,11,15-tetraen-3-ol	27.31	2.73	270, 135, 121, 81
13	Thunbergol	30.84	1.13	272, 229, 147, 81
14	Geranylgeraniol	28.47	6.21	272, 257, 93, 69
15	Loliolide	22.98	1.45	178, 140, 111, 43
	**Steroids**			
16	Cholesterol	38.15	0.42	368, 353, 301, 275
17	Ergosta-5,24-dien-3β-ol	40.47	3.89	383, 365, 311, 299
18	22,23-Dibromostigmasterol acetate		0.09	554, 394, 255, 213
19	Androstane-3,17-dione	25.40	0.66	270, 244, 217, 199
20	Gorgosterol	46.49	1.18	408, 355, 337, 314
	**Fatty acids and their derivatives**			
21	2,5,10-Undecatrienoic acid methyl ester	30.58	1.62	179, 152, 95, 79
22	5,8,11,14,17-Eicosapentaenoic acid methyl ester	27.81	3.02	301, 287, 245, 95
23	Arachidonic acid	27.63	2.32	280, 265, 119, 79
24	11-Hexadecenoic acid	24.24	3.42	239, 222, 115, 73
25	Methyl palmitoleate	24.05	0.32	222, 180, 143, 87
26	Methyl palmitate	27.81	3.32	227, 185, 143, 74
27	Methyl stearate	26.68	2.12	283, 267, 227, 74
28	Tetradecyl octadecanoate	23.28	0.28	438, 286, 241, 196
29	Methyl tetradecanoate	22.59	0.16	227, 211, 185, 74
	**Nitrogenous derivatives**			
30	D-Methionine	18.93	0.26	131, 101, 83, 61
31	2-Aminopurine	22.36	0.90	135, 108, 81, 54
32	1-(4-bromobutyl)-2-piperidinone	36.16	0.46	205, 154, 97, 69
33	1,3-bis(4-methoxyfurazan-3-yl)-3-propyl-triazene	21.25	0.63	247, 230, 210, 127

Area % = relative abundance; compound identification was achieved by analyzing retention times, mass spectra, and fragmentation patterns, which were then compared to standard reference data from the Wiley and NIST libraries.

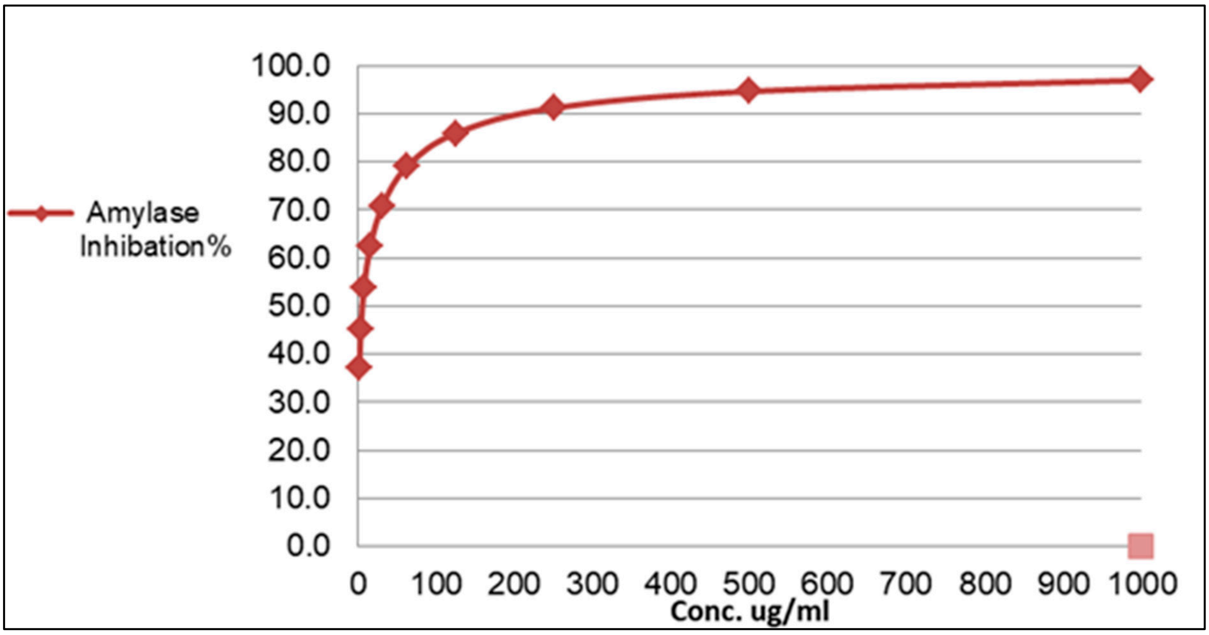

Figure S1. Dose–response curve of α-amylase inhibition by an alcoholic extract of *Tripneustes gratilla*. Values are presented as mean % inhibition ± SEM (n=3).

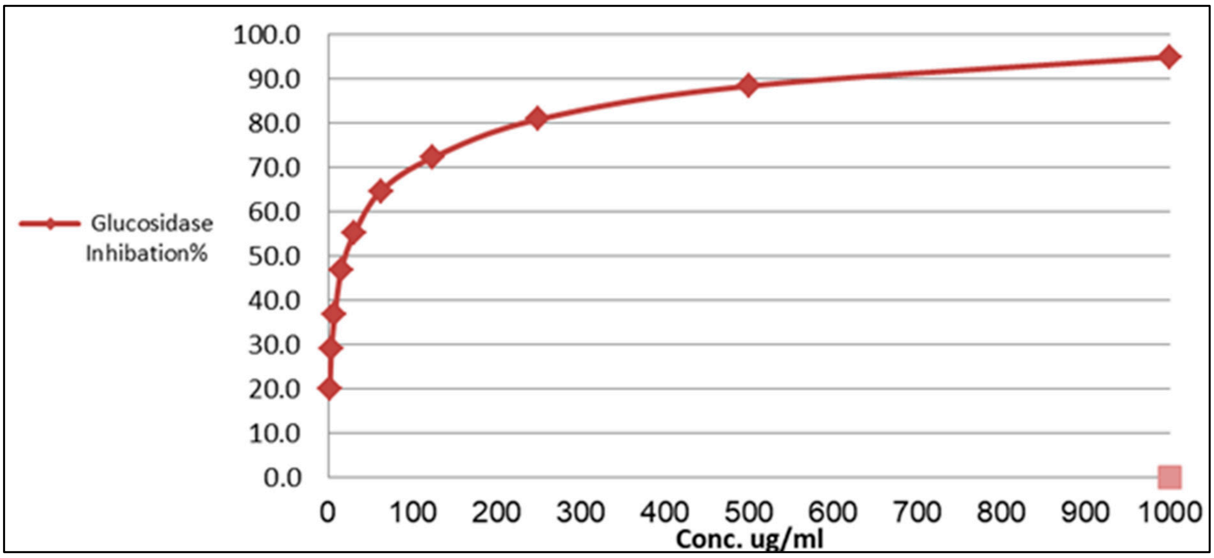

Figure S2. Dose–response curve of α-Glucosidase inhibition by an alcoholic extract of *Tripneustes gratilla*. Values are presented as mean % inhibition ± SEM (n=3).

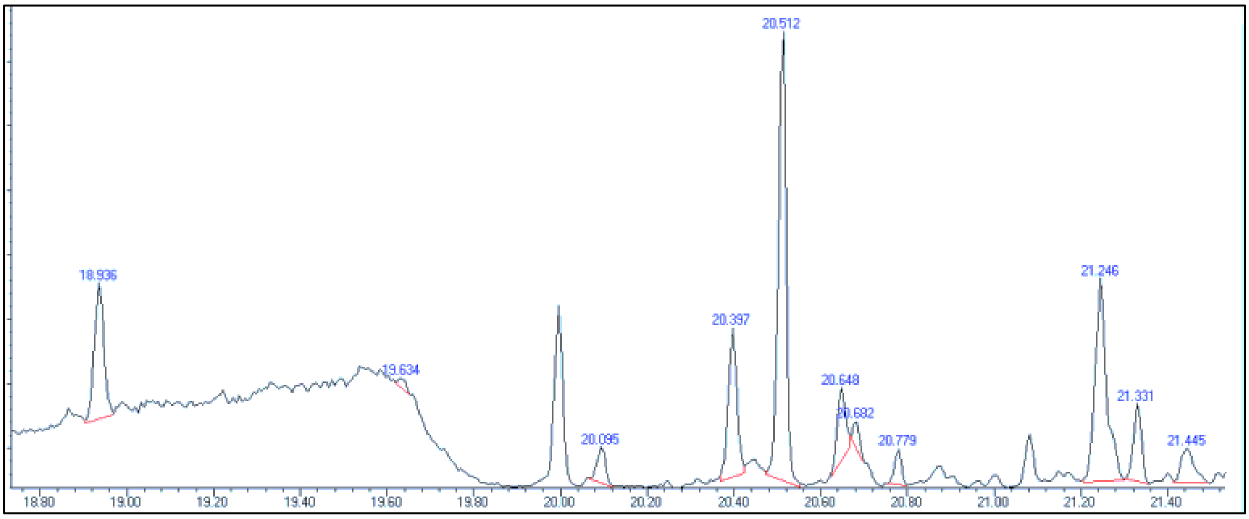

Figure S3. Expanded GC-chromatograms of alcoholic extract of *Tripenasteus gratilla* (retention time 18.8- 21.4).

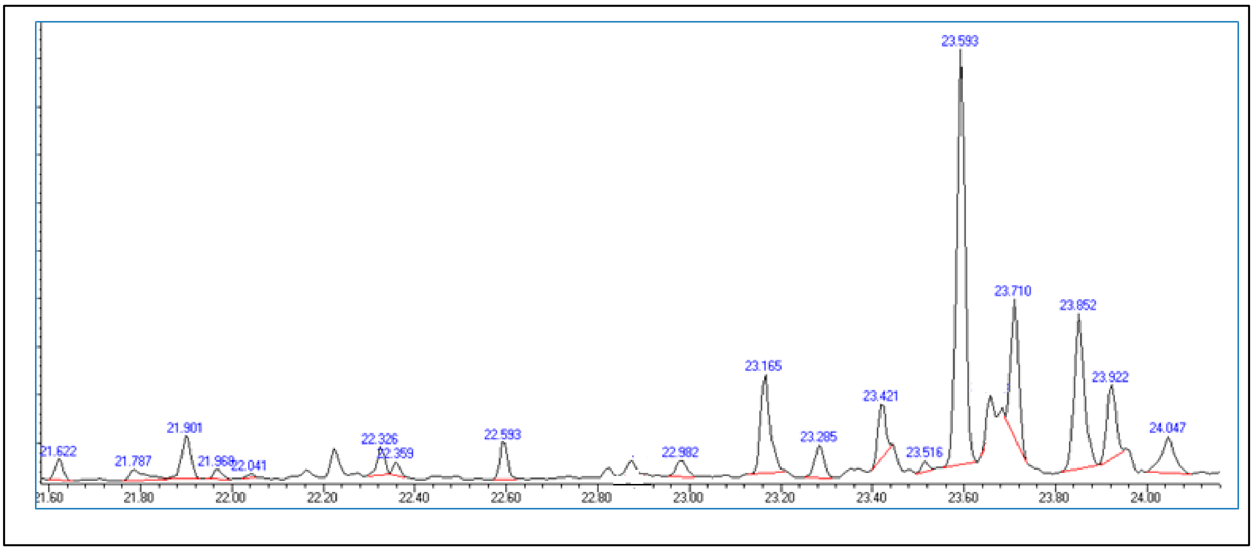

Figure S4. Expanded GC-chromatogram of alcoholic extract of *Tripenasteus gratilla* (retention time 21.6-24.0).

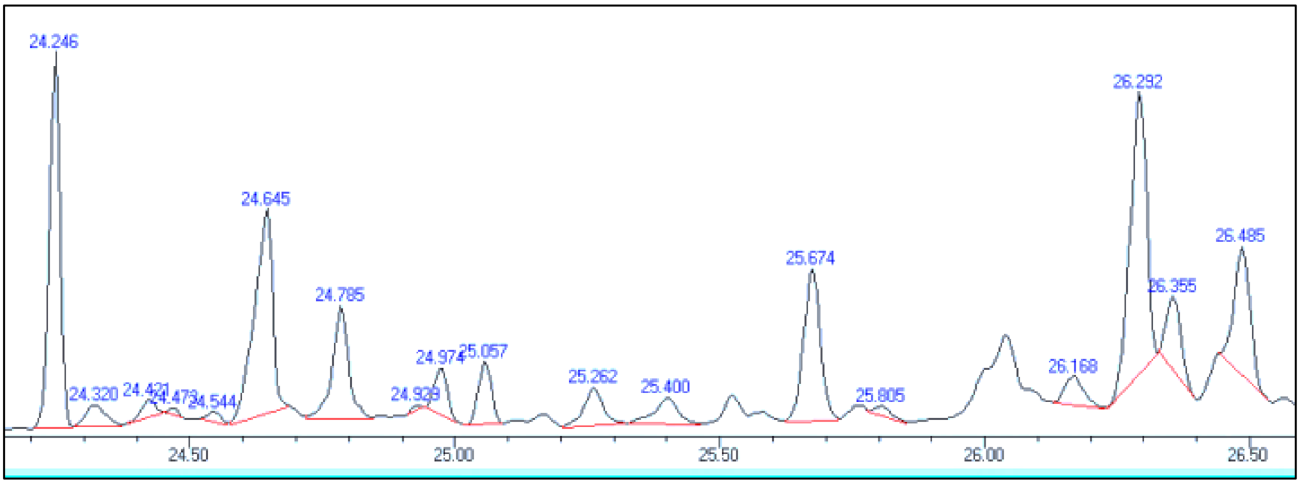

Figure S5. Expanded GC-chromatogram of alcoholic extract of *Tripenasteus gratilla* (retention time 24.2- 26.5).

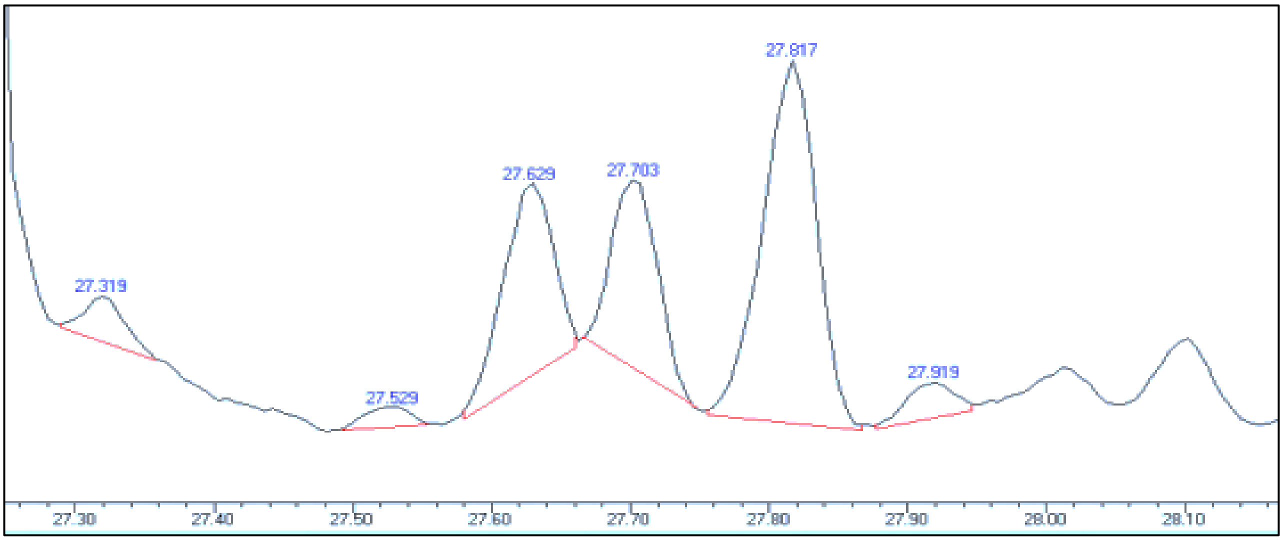

Figure S6. Expanded GC-chromatogram of alcoholic extract of *Tripenasteus gratilla* (retention time 27.2- 28.1).

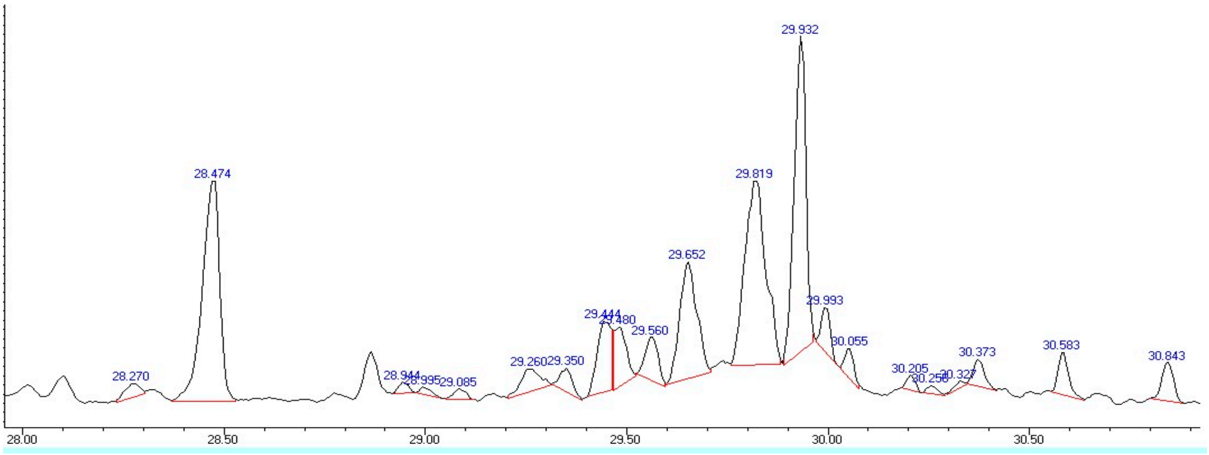

Figure S7. Expanded GC-chromatogram of alcoholic extract of *Tripenasteus gratilla* **(**retention time 28.0- 31.0).

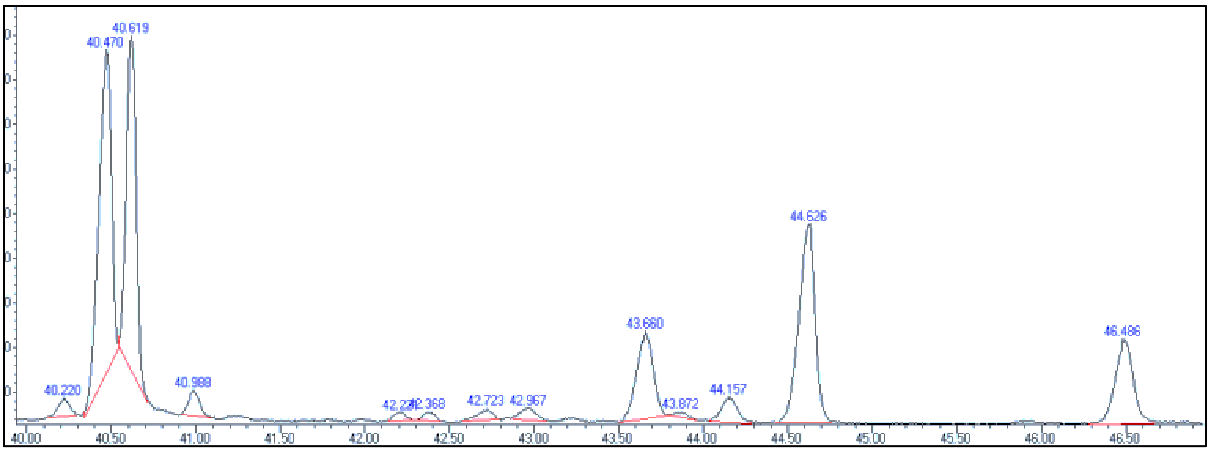

Figure S8. Expanded GC-chromatogram of alcoholic extract of *Tripenasteus gratilla* (retention time 40.0- 46.5).

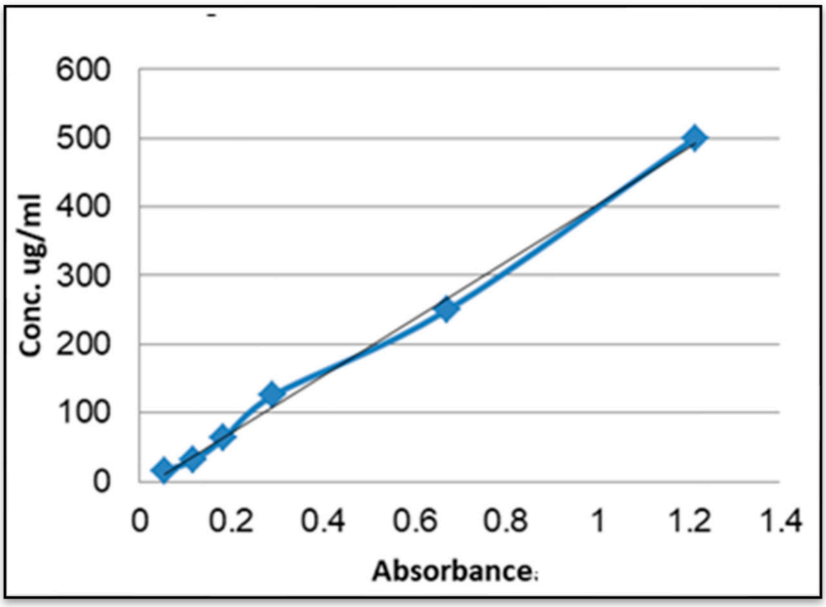

Figure S9. Calibration curve of gallic acid (standard) used for the quantification of total phenolic content (TPC) in the alcoholic extract of *T. gratilla*. The regression equation was y = 415.4x − 11.236 with a correlation coefficient $R^2 = 0.996$.
